# Variation in lanternfish (Myctophidae) photophore structure: A comprehensive comparative analysis

**DOI:** 10.1371/journal.pone.0310976

**Published:** 2024-11-13

**Authors:** Rene P. Martin, Emily M. Carr, John S. Sparks

**Affiliations:** 1 Department of Ichthyology, Division of Vertebrate Zoology, American Museum of Natural History, New York, New York, United States of America; 2 University of Nebraska-Lincoln, School of Natural Resources, Lincoln, Nebraska, United States of America; 3 Richard Gilder Graduate School, American Museum of Natural History, New York, New York, United States of America; Nanjing Agricultural University, CHINA

## Abstract

The deep-sea open ocean habitat (below 200 m depth) is comprised of little-to-no light, near freezing temperatures, and vastly connected stratified waters. Bioluminescence is often linked to the success and diversification of fishes in these dark deep-sea habitats, which are host to many species-rich and morphologically diverse clades. Fish bioluminescence takes many forms and is used in a variety of behaviors including counterillumination, prey detection and luring, communication, and predator avoidance. This study focuses on lanternfishes (Myctophidae), a diverse group (252 spp. in 34 genera) of deep-sea fishes in which bioluminescence has played a critical role in their diversification. Using histological techniques, we provide new morphological analyses of the complex structure of the primary photophores of representative species from 17 genera in which photophore morphology has not previously been described. We combine this information with data from prior studies to compare primary photophore characteristics for species representing all 34 lanternfish genera. Although we find that lanternfish primary photophores are similar in many of their structural components, including the possession of a modified scale cup, photocytes, pigment, and reflector layers, we observe significant variation among species in other aspects of photophore morphology. Observed morphological differences include variation in pigmentation and in the calcification and thickness of the modified scale cup. We also find reflectors that are very thin or absent in gymnoscopeline and lampanyctine species, relative to the robust reflectors present in myctophine species. We find evidence of secondary reflectors and secondary pigment layers in six lanternfish species and observe major differences in scale-lens thickness and mineralization across the assemblage. Lastly, *Scopelopsis multipunctatus* is the only species analyzed lacking a photophore cup. Obtaining finer detail of light organ morphology across this species-rich lineage provides much-needed insight into the factors that have contributed to the remarkable diversity of lanternfishes in the deep open ocean.

## Introduction

The deep sea (oceanic water depths below 200 m) is an under-explored area compared to near-shore marine systems. It is host to extreme abiotic factors including little-to-no light, nearly freezing temperatures, and intense hydrostatic pressures [1–3]. The combined characteristics of these abiotic factors makes it difficult to observe, sample, and preserve specimens from the deep sea in their natural state compared to diverse freshwater or near-shore marine groups [4]. Further limitations of current sampling methods, like trawling at low speeds and through limited depth profiles, hinders accurate assessments of mesopelagic or deep-sea fish biodiversity and can have deleterious effects on specimens collected. Thus, studies focused on morphological diversity across deep-sea fish lineages are lacking despite the multitude of species-rich [5,6] and morphologically diverse [7] clades. The specific selective pressures of the deep sea have resulted in an array of specializations common to many deep-sea lineages, including fangs, dorsally directed eyes, ultra-black pigmentation, and bioluminescence [8–11]. Bioluminescence is believed to be a key innovation to the success and diversification of many shallow water and deep-sea fishes [12–15]. This phenomenon occurs when a luciferin molecule is oxidized by an enzyme (luciferase) in the presence of oxygen [8]. Bioluminescent structures take many forms in fishes; including ventral photophores used in counterillumination, head-associated light organs and chin barbels used for prey detection and luring. Some species exhibit patches of bioluminescent tissues on the body used in communication and recognition of conspecifics while others possess pouches that secrete bioluminescent fluid used to distract predators. Lastly, many produce bioluminescence via anatomically complex internal structures that house symbiotic bioluminescent bacteria [12–14,16,17].

This study focuses on one of the most species-rich groups of bioluminescent deep-sea fishes, the lanternfishes (Myctophidae), with 252 valid species arrayed in 34 genera [4,14]. Lanternfishes are in the order Myctophiformes and are sister to the relatively species depauperate (6 spp. in 3 genera) blackchins (Neoscopelidae) [4,18]. Myctophidae is currently comprised of five subfamilies, including Diaphinae (82 spp.), Gymnoscopelinae (18 spp.), Lampanyctinae (74 spp.), Myctophinae (77 spp.), and Notolychninae (1 sp.) [18]. Lanternfishes are small and extremely abundant deep-sea mesopelagic (200–1000 m) fishes, representing the majority of fish biomass recovered in mesopelagic trawls [19]. Lanternfishes are believed to have an increased rate of diversification relative to other deep-sea lineages [14], surprising given the relatively stable environment of the deep sea, with no obvious barriers to gene flow and a distinct lack of abiotic isolating mechanisms. Researchers have speculated on how lanternfishes, occurring worldwide and in such extreme abundance, have diversified to such an extent [20]; and one of the most compelling hypotheses involves bioluminescent signaling [14,21,22].

**Fig 1:**
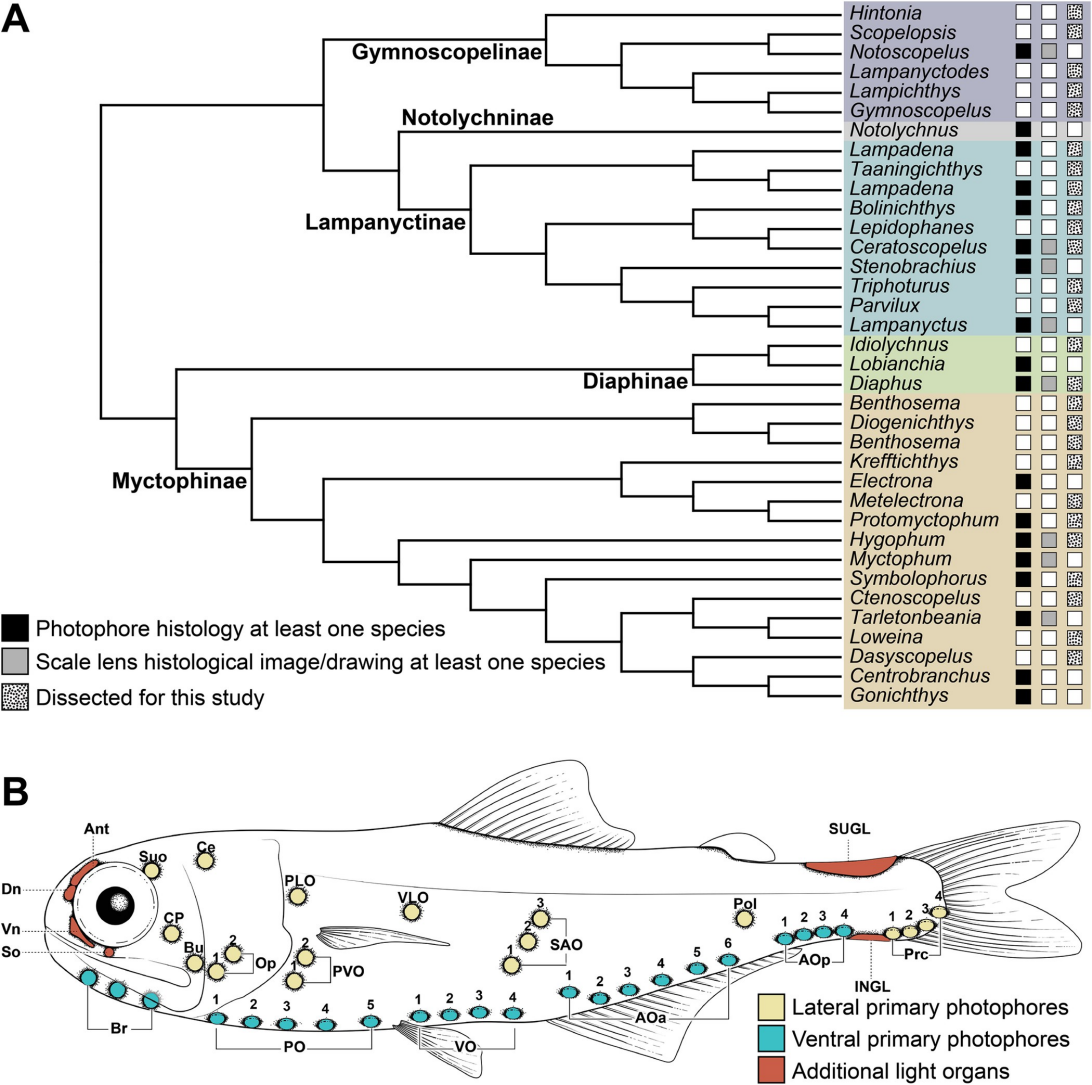
Phylogenetic relationships of lanternfishes and illustration of common light organs and primary photophores. (A) Lanternfish genus-level cladogram from Martin et al. (2018) illustrating genera with previous research investigating cellular structure of primary photophores. A list of associated studies can be found in text. (B) Rendering of a generalized lanternfish depicting primary photophore series and additional common light organs. Abbreviations are as follows: Ant, antorbital organ; AOa, anterior anal organs; AOp, posterior anal organs; Br, branchiostegal organs; Bu, buccal organ; Ce, cervical; CP, cheek photophore; Dn, dorsonasal organ; INGL, infracaudal luminous gland; Op, opercular organs; PLO, suprapectoral organ; PO, pectoral organs; Pol, postero-lateral organ; Prc, precaudal organs; PVO, subpectoral luminous glands; SAO, supraanal organs; So, suborbital organ; Suo, supraorbital organ; SUGL, supracaudal luminous gland; VLO, supraventral organ; Vn, ventronasal organ; VO, ventral organs.

**Fig 2:**
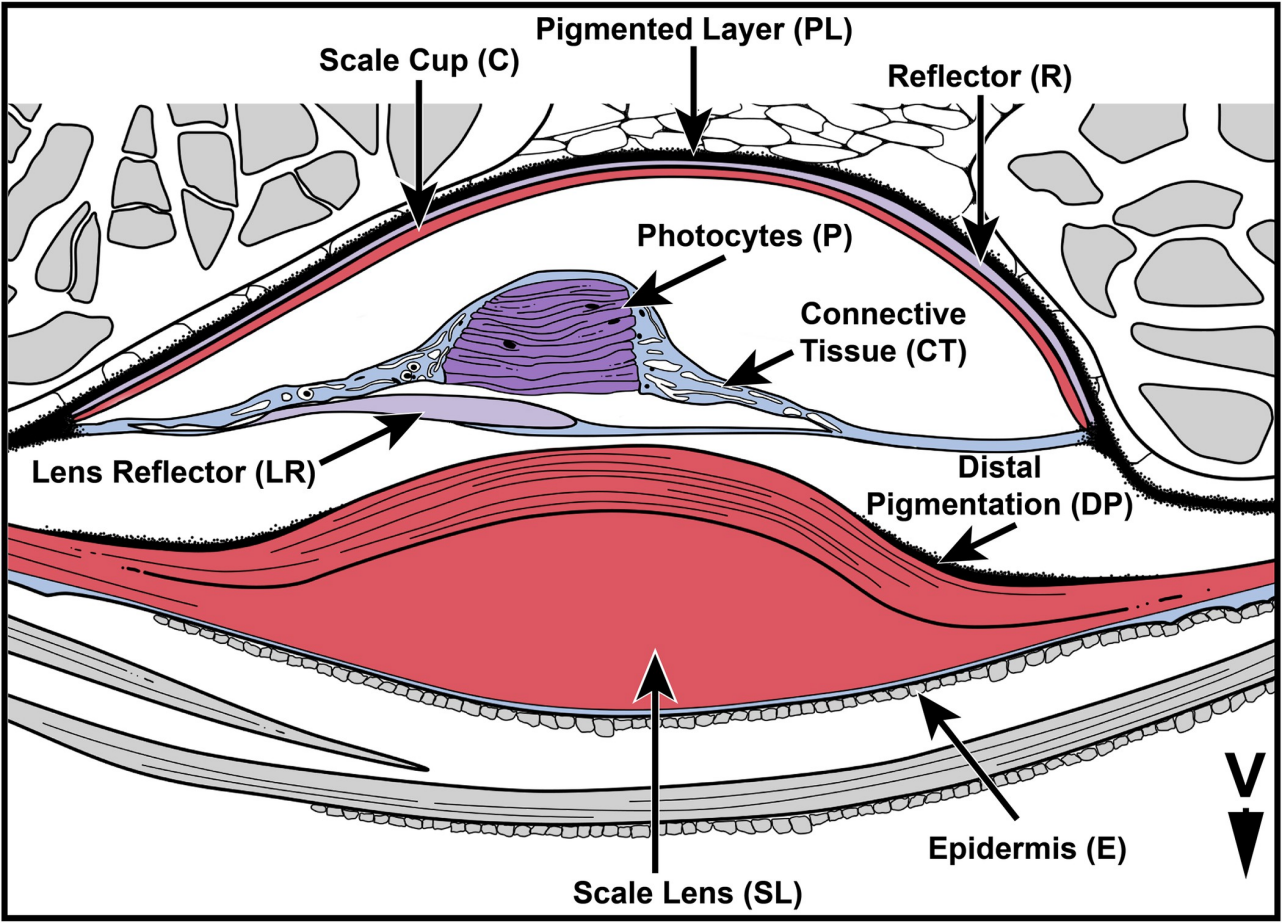
Generalized illustration of the lateral section through a lanternfish VO primary photophore. Main elements colored similar to tissues stained via Masson’s trichrome stain. Arrow V points ventrally.

Lanternfishes possess intrinsic (non-bacterial) bioluminescence. Their light organs fall into two categories: 1) primary photophores, structures usually associated with a reflective modified scale cup and scale lens, and 2) additional light organs and luminescent patches of bioluminescent tissue not associated with a scale cup or lens (Figs 1B and 2). All but one species of lanternfish, *Taaningichthys paurolychnus*, possess primary photophores located either ventrally (Fig 1B: teal photophores) or laterally (Fig 1B: yellow photophores) on the body [21,23,24]. Ventral primary photophores are used in camouflage via counterillumination, where emitted light matches the downwelling light from the surface of the ocean, effectively obscuring the silhouette. The positions of ventral primary photophores are believed to be ecologically constrained and are not useful for discriminating between species [14]. However, lateral primary photophores are hypothesized to be used in communication and are highly species specific and phylogenetically informative [14,21,23,24]. The additional light organs found on lanternfishes (Fig 1B: orange) generally occur on the head (head-light) or caudal peduncle (tail-light), but can also be present on other parts of the body (e.g., patches at the pectoral-fin insertion, along the dorsal or ventral margins of the body). The head-light and tail-light organs of many lanternfishes are sexually dimorphic and thought to be used in intra- and interspecific signaling [17,25–27].

Unfortunately, few studies have focused on the morphological and histological variation of lanternfish primary photophores, the associated scale lens, and additional light organs (Fig 1) [28–34]. These anatomically complex organs (Fig 2) produce light via innervated multinucleated photocytes [29]. Luminescence from the photocytes (P) is prevented from being emitted at unwanted angles by lens reflectors (LR). These reflectors are associated with the thickened modified scale lens (SL) and are located distal to the photocytes (Fig 2). Emitted and reflected light is directed onto a parabola-like reflector (R) comprised of hexagonal-shaped guanine iridophores. This reflector is overlayed by a scale cup (C), which is hypothesized to be a modified scale (Fig 2) [31,33]. As a result, reflected light is emitted in a specific direction through the ‘photophore aperture’ or the thickened scale lens area [29,31,33]. Of the 252 lanternfish species, histological work has been performed on the primary photophores of only 33 species, representing 17 of the 34 genera (~13% of species diversity). Research on 23 of these species was performed in the late 1800’s to early 1900’s [35–41] and studies on the remaining 10 have occurred over the last seven decades [28–34,42,43]. Very few of these works include detailed descriptions of variation in cellular structure by species, even less include accompanying drawings, and most lack any associated images of cell morphology. Of the aforementioned studies, even fewer note [29,32] or assess the composition of the scale lens [30,42], which is an integral structure of the primary photophores of lanternfishes. Although there are a limited number of studies to date, considerable variation in the ultrastructure of lanternfish photophores has been reported [28–34]. This highlights the need for a more widespread and taxonomically comprehensive survey across the lineage to better understand the extent of light-organ structural variation in the anatomically complex primary photophores of lanternfishes.

In this study, primary photophore anatomy and morphology from all newly assessed genera is incorporated with information on primary photophores reported in previously published works to achieve a greater understanding of primary photophore structure across Myctophidae, including representatives from all 34 genera. The goals of this study are as follows: 1) perform histological analyses on the primary photophores of lanternfish species from previously uninvestigated genera, 2) analyze the structure and ultrastructure of primary photophores and their associated scale lens across all lanternfish genera incorporating information from previous studies on primary photophore morphology, and 3) describe similarities and differences in the morphology of primary photophores across Myctophidae.

## Materials and methods

### Dissected specimens and histology

Histological analyses for the anatomical description of primary photophores included 24 species (25 specimens) representing 24 of the 34 myctophid genera. The primary photophores of 17 of these genera have not been analyzed in previous studies (Fig 1A; Table 1). Lanternfishes often lose their scales and skin during collection. When possible, specimens were selected that possessed visible primary photophores in good condition and with intact scales. Specimens used in this study were housed at the American Museum of Natural History (AMNH) or were requested on loan from the Museum of Comparative Zoology (MCZ) and the Natural History Museum of Los Angeles County (LACM).

**Table 1:** Lanternfish specimens dissected for histological analysis in this study.

Species	Museum ID	SL (mm)	Scale Lens Thickness (μm)	Fig
*Benthosema suborbitale*	AMNH 270136	36	136.1	6A
*Bolinichthys longipes*	AMNH 266513	33	56.0	4A
*Ceratoscopelus townsendi*	AMNH 277131	48	39.0	4B
*Ctenoscopelus phengodes*	LACM 31317–7	24	55.8	6B
*Dasyscopelus obtusirostris*	AMNH 30537	34	43.8	6C
*Diaphus regani*	AMNH 270350	31	-	5A
*Diogenichthys atlanticus*	MCZ 117647	19	-	6D
*Gymnoscopelus braueri*	MCZ 1148790	90	73.9	3A
*Hintonia candens*	LACM 11329–3	22	-	3B
*Hygophum proximum*	AMNH 270116	45	120.0	6E
*Idiolychnus urolampus*	LACM 37002–1	87	-	5B
*Krefftichthys anderssoni*	LACM 11494–12	27	-	7A
*Lampadena urophaos*	AMNH 261300	38	-	4D
*Lampanyctodes hectoris*	LACM 10969–6	45	-	3C
*Lampichthys procerus*	MCZ 102795	33		3D
*Lepidophanes guentheri*	AMNH 258059	53	-	4C
*Loweina rara*	LACM 38251–3	36	50.7	7B
*Metelectrona ventralis*	LACM 11077–13	28	-	7C
*Parvilux ingens*	LACM 9559–12	43	-	4E
*Protomyctophum arcticum*	AMNH 215743	40		7D
*Scopelopsis multipunctatus*	MCZ 102573	54	27.7	3E
*Scopelopsis multipunctatus*	LACM 31380–7	50	-	3E
*Symbolophorus evermanni*	AMNH 270083	62	-	7E
*Taaningichthys minimus*	AMNH 270079	55	-	4F
*Triphoturus mexicanus*	AMNH 252051	50	74.0	4G

For histological sectioning, a piece of epithelial and muscle tissue approximately 0.5–1 cm^3^ was removed containing the first 1–4 right-side primary photophores of the ventral organ series (VO; Fig 1). These tissues were removed from previously formaldehyde-fixed and ethanol preserved lanternfish specimens while using a Nikon SMZ800N stereo microscope. Photophores from the VO series were chosen because their scale-lens’ are often still present, as the pelvic fins provide protection against scale loss during collection. Thus, if present, the modified scale lens and associated scales overlaying the primary photophores were also removed with the tissue. Dissected epithelia containing embedded photophores and scales (when present) were placed into 70% EtOH. Samples were decalcified in a citrate buffered 25% folic acid solution for 15–20 minutes followed by dehydration through a graded ethanol series up to 100%. Samples were cleared in xylene followed by infiltration and embedding in paraffin wax. Primary photophores were cut in serial sections at 10μm thickness in the transverse plane using a Leica HistoCore MULTICUT microtome. Sections were floated on a 45°C water bath, mounted on charged glass slides, and placed on a slide warmer set at 48°C until dry. Dried sections were deparaffinized in xylene and stained using either Masson’s trichrome (MT) following the protocol from Ghedotti et al. [44] to differentiate collagen and muscle [45] or Toluidine Blue (TB). Stained sections were then mounted in Canada balsam or Permount. Histological preparations were conducted at the American Museum of Natural History, New York, NY and at Regis University, Denver, CO. Stained and mounted sections were examined under a Nikon Eclipse 50*i* compound microscope and photographed using an attached Excelis 4K UHD microscope camera. Photos were prepared for figures by increasing brightness and contrast, by eliminating debris and discoloration that occurred during the staining and mounting process, and by combining photographs from higher magnifications into composite images using Adobe Photoshop.

### Previously published information

Previously published information on the anatomy and morphology of lanternfish primary photophores is available for 33 species representing 17 of the 34 genera. These works, in addition to newly analyzed specimens from this study, comprise 40 species and all 34 lanternfish genera (Fig 1). Comparative analyses across Myctophidae were informed by material and analyses from the following studies: Leydig [35], Emery [36], Gatti [38], Lendenfeld [39], Brauer [40], Ohshima [41], Nicol [28], Iwai and Okamura [42], O’Day [29], Lawry [30], Denton et al. [31], Cavallaro et al. [32,34], and Paitio et al. [33]. A comprehensive list of all comparative lanternfish species used in this study (including those newly analyzed), the morphological features of their photophores, and their associated literature references can be found in Table 2.

**Table 2:** All lanternfish species assessed in this study including newly dissected (see additional Table 1) and those described and analyzed in previous publications.

Subfamily	Species	Reference	(C)	(P)	(PL)	(R)	(SR)	(SPL)	(SL) Sectioned
Diaphinae	*Diaphus dumerilii*	Brauer 1908 [40]	X	X	X	X	-	-	X
	*Diaphus rafinesquii*	Leydig 1881 [35]	X	X	X	X	-	-	X
	Diaphus regani*	This Study	X	-	X	-	-	-	-
	*Diaphus watasei*	Paitio et al. 2020 [33]	X	X	X	X	-	-	X
	*Idiolychnus urolampus*	This Study	X	X	X	X	-	-	-
	*Lobianchia*	Gatti 1904 [38]	X	X	X	X	-	-	-
Gymnoscopelinae	*Gymnoscopelus braueri*	This Study	X	X	X	X	-	-	X
	Hintonia candens*	This Study	X?	X?	-	-	-	-	-
	Lampanyctodes hectoris*	This Study	X	X	-	-	-	-	-
	*Lampichthys procerus*	This Study	X	X	X	-	-	-	-
	*Notoscopelus elongatus*	Emery 1884 [36]	X	X	X	X	-	-	X
	*Scopelopsis multipunctatus*	This Study	-	X	X	-	-	-	X
Lampanyctinae	*Bolinichthys longipes*	Brauer 1908 [40]	X	X	X	-	-	-	X
	*Bolinichthys longipes*	This Study	X	X	X	-	-	-	X
	*Ceratoscopelus maderensis*	Cavallaro et al. 2019 [32]	X	X	X	X	-	-	X
	Ceratoscopelus townsendi*	This Study	X	-	X	-	-	-	X
	*Lampadena urophaos*	This Study	X	-	X	-	-	-	-
	*Lampanyctus macropterus*	Brauer 1908 [40]	X	X	X	-	-	-	X
	*Lepidophanes guentheri*	This Study	X	X	X	-	-	-	-
	*Parvilux ingens*	This Study	X	X	X	-	-	X	-
	*Stenobrachius leucopsarus*	O’Day 1972 [29]	X	X	X	X	X	X	X
	*Taaningichthys minimus*	This Study	X	X	X	-	-	-	-
	*Triphoturus mexicanus*	This Study	X	X	X	-	-	X	X
Myctophinae	*Benthosema suborbitale*	This Study	X	X	X	X	X	X	X
	*Centrobranchus andreae*	Brauer 1908 [40]	X	X	X	X	-	-	-
	*Ctenoscopelus phengodes*	This Study	X	X	X	-	-	-	X
	*Dasyscopelus obtusirostris*	This Study	X	X	X	X	-	-	X
	Diogenichthys atlanticus*	This Study	X	X	X	-	-	-	-
	*Electrona rissoi*	Leydig 1881 [35]	X	X	X	X	-	-	X
	*Electrona rissoi*	Emery 1884 [36]	X	X	X	X	-	-	X
	*Gonichthys coccoi*	Brauer 1908 [40]	X	X	X	X	-	-	-
	*Hygophum benoiti*	Brauer 1908 [40]	X	X	X	X	-	-	X
	*Hygophum proxiumum*	This Study	X	X	X	X	-	-	X
	*Krefftichthys anderssoni*	This Study	X	X	X	X	-	-	-
	*Loweina rara*	This Study	X	X	X	X	X	X	X
	*Metelectrona ventralis*	This Study	X	X	X	X	-	-	-
	*Myctophum aurolaternatum*	Lendenfeld 1905 [39]	X	X	X	X	-	-	X
	*Myctophum punctatum*	Leydig 1881 [35]	X	X	X	X	-	-	X
	*Protomyctophum arcticum*	This Study	X	X	X	X	-	-	-
	*Symbolophorus evermanni*	This Study	X	X	X	X	X	X	-
	*Tarletonbeania crenularis*	Lawry 1973 [30]	X	X	X	X	-	-	X
Notolychninae	*Notolychnus valdiviae*	Brauer 1908 [40]	X	X	X	X	-	-	-

Stars next to species names denote specimens that were poorly preserved. Presence of a structure indicated with an X. Dashes indicate structure not observed or not mentioned in previous studies. Abbreviations are as follows: C, photophore cup; P, photocytes, PL, main pigment layer; R, main reflector layer; SR, secondary reflector layer; SPL, secondary pigment layer; SL, scale lens.

## Results

### Primary photophore structure

**Fig 3:**
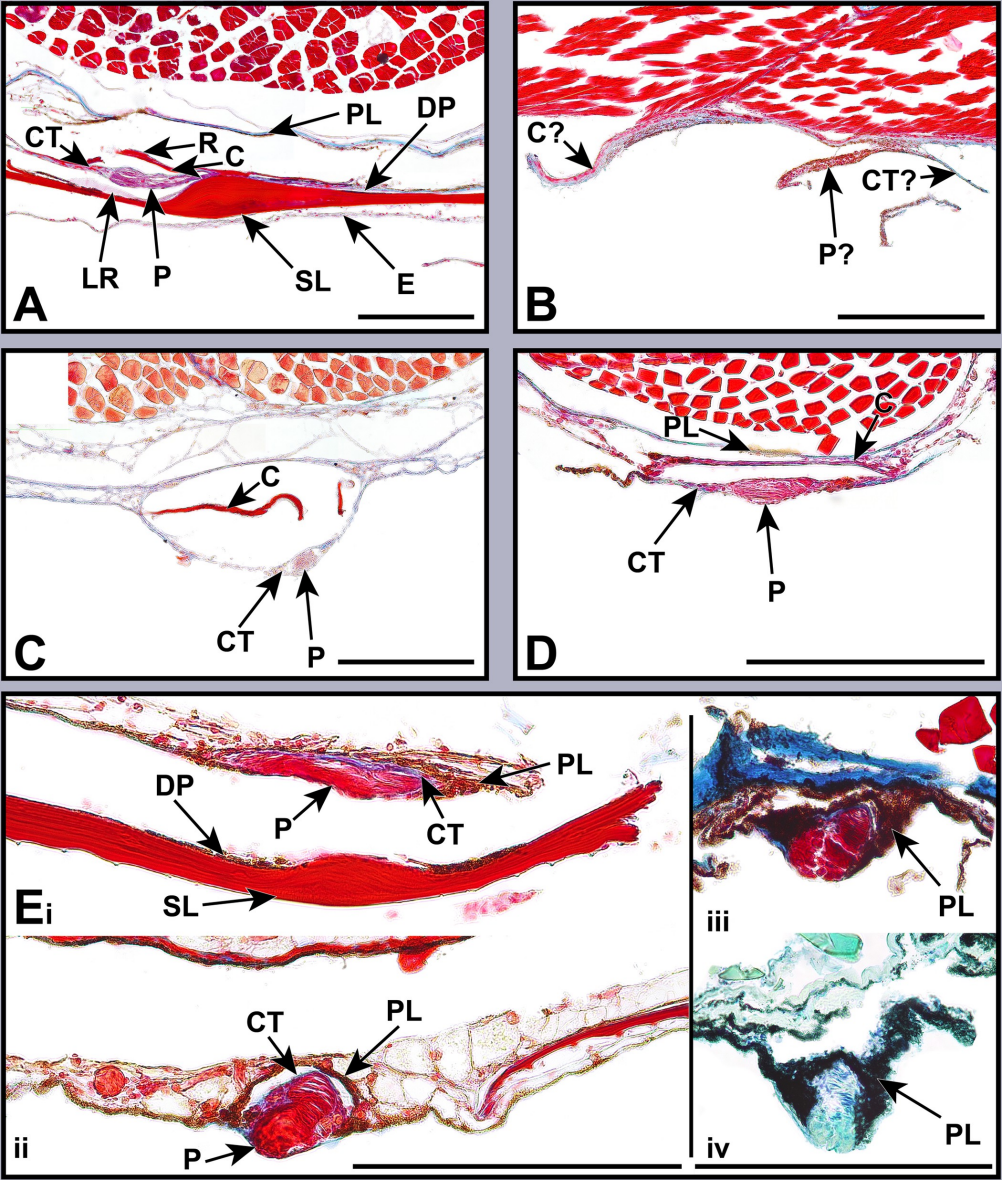
Stained sections of lanternfish primary VO photophores from species in the subfamily Gymnoscopelinae. Organized alphabetically by genus. Masson’s trichrome: (A) *Gymnoscopelus braueri* MCZ 148790. (B) *Hintonia candens* LACM 11329–3. (C) *Lampanyctodes hectoris* LACM 10969–6. (D) *Lampichthys procerus* MCZ 102795. (E_i_, E_ii_) *Scopelopsis multipunctatus* MCZ 102573. (E_iii_) *Scopelopsis multipunctatus* LACM 31380–7. Toluidine blue: (E_iv_) *Scopelopsis multipunctatus* LACM 31380–7. Abbreviations are as follows: C, scale cup; CT, connective tissue; DP, distal pigmentation; E, epidermis; LR, lens reflector; P, photocytes; PL, pigmented layer; R, reflector; SL, scale lens. Scale bars represent 200 μm.

**Fig 4:**
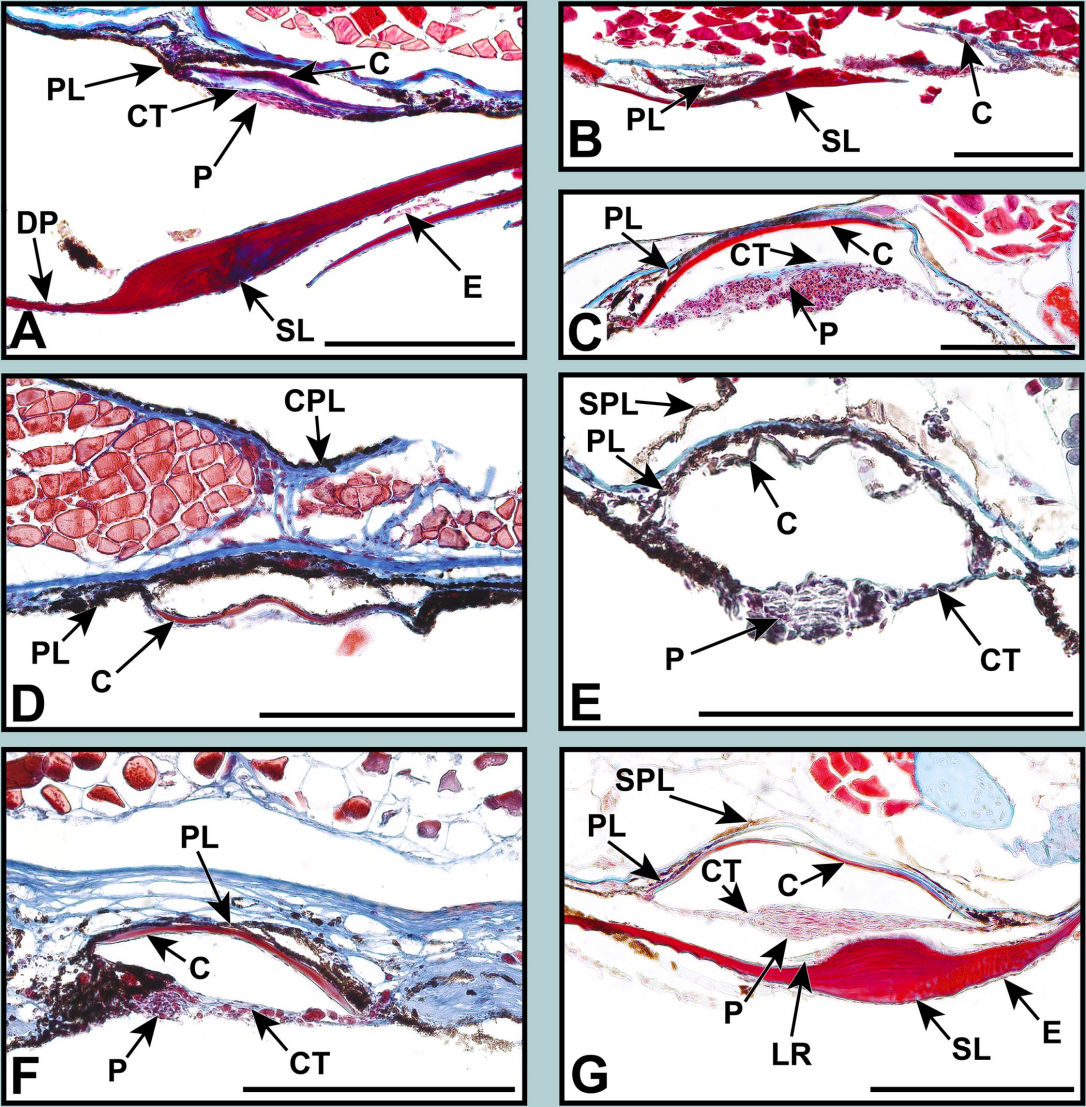
Stained sections of lanternfish primary VO photophores from species in the subfamily Lampanyctinae. Masson’s trichrome: (A) *Bolinichthys longipes* AMNH 266513. (B) *Ceratoscopelus townsendi* AMNH 277131. (C) *Lepidophanes guentheri* AMNH 258059. (D) *Lampadena urophaos* AMNH 261300. (E) *Parvilux ingens* LACM 9559–12. (F) *Taaningichthys minimus* AMNH 270079. (G) *Triphoturus mexicanus* AMNH 252051. Abbreviations are as follows: C, cup; CPL, coelom pigment layer; CT, connective tissue; DP, distal pigmentation; E, epidermis; LR, lens reflector; P, photocytes; PL, pigmented layer; R, reflector; SL, scale lens; SPL, secondary pigment layer. Scale bars represent 200 μm.

**Fig 5:**
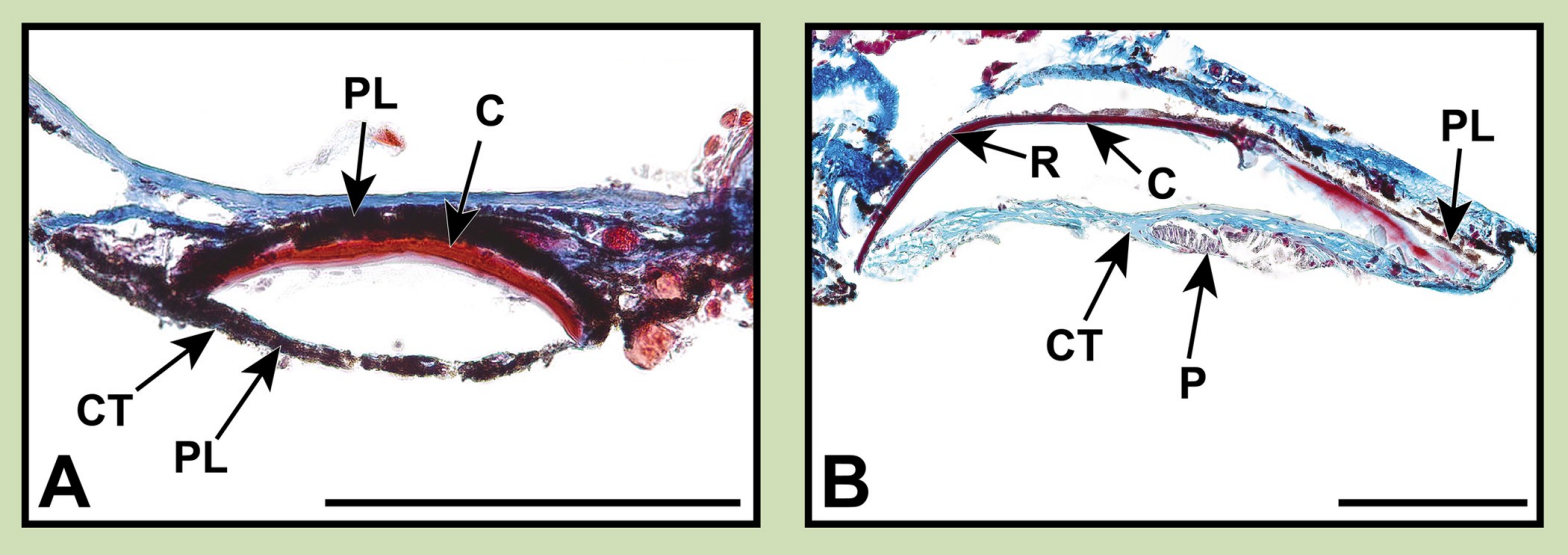
Stained sections of lanternfish primary VO photophores from species in the subfamily Diaphinae. Organized alphabetically by genus. Masson’s trichrome: (A) *Diaphus regani* AMNH 270350. (B) *Idiolychnus urolampus* LACM 37002–1. Abbreviations are as follows: C, cup; CT, connective tissue; P, photocytes; PL, pigmented layer; R, reflector. Scale bars represent 200 μm.

**Fig 6:**
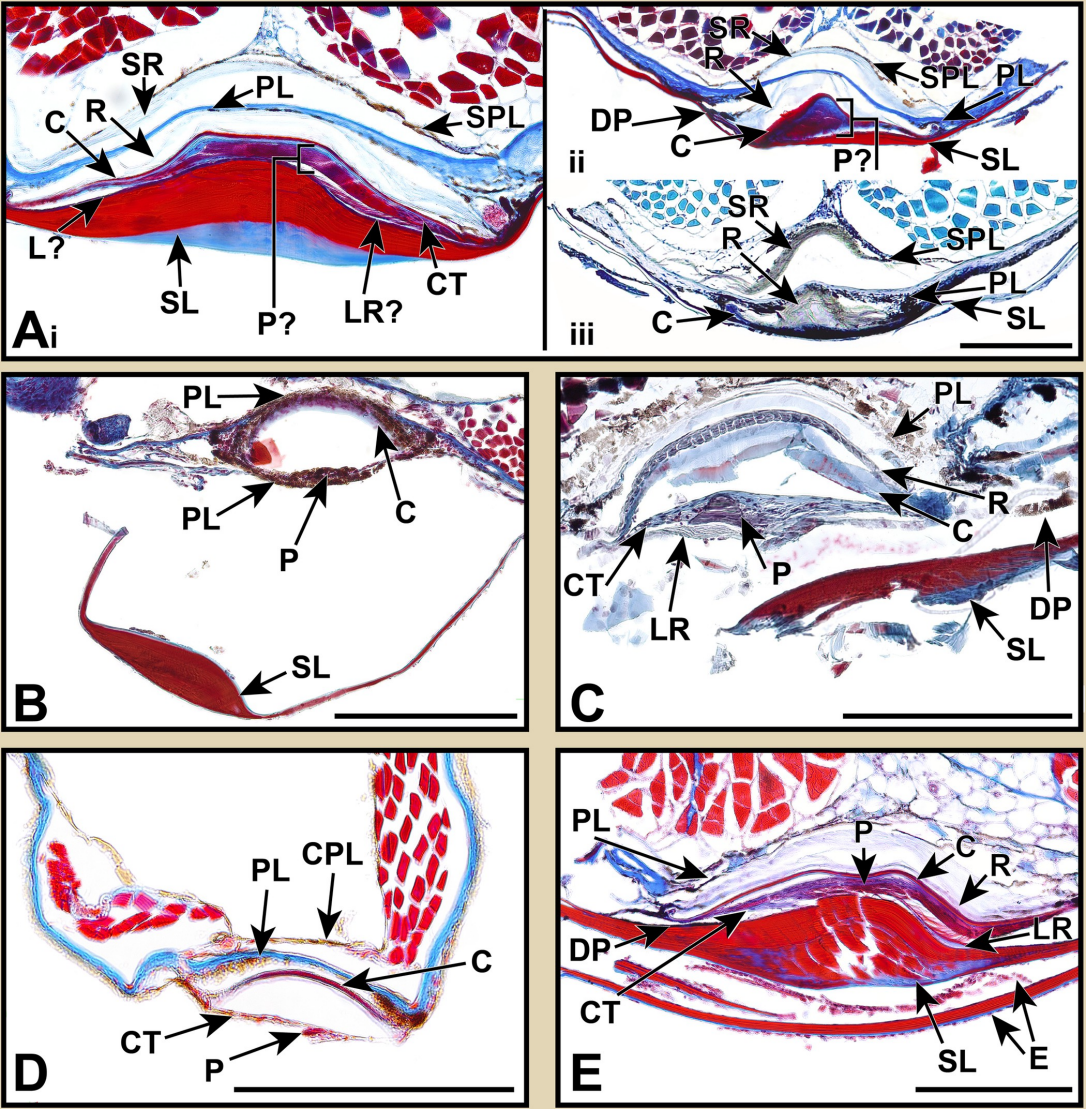
Stained sections of lanternfish primary VO photophores from species in the subfamily Myctophinae. Organized alphabetically by genus. Masson’s trichrome: (A_i_, _ii_) *Benthosema suborbitale* AMNH 270136. (B) *Ctenoscopelus phengodes* LACM 31317–7. (C) *Dasyscopelus obtusirostris* AMNH 30537. (D) *Diogenichthys atlanticus* MCZ 117647. (E) *Hygophum proximum* AMNH 270116. Toluidine blue: (A_iii_) *Benthosema suborbitale* AMNH 270136. Abbreviations are as follows: C, cup; CT, connective tissue; DP, distal pigmentation; E, epidermis; L?, unknown layer; LR, lens reflector; P, photocytes; PL, pigmented layer; R, reflector; SL, scale lens; SPL, secondary pigment layer; SR, secondary reflector. Scale bars represent 200 μm.

**Fig 7:**
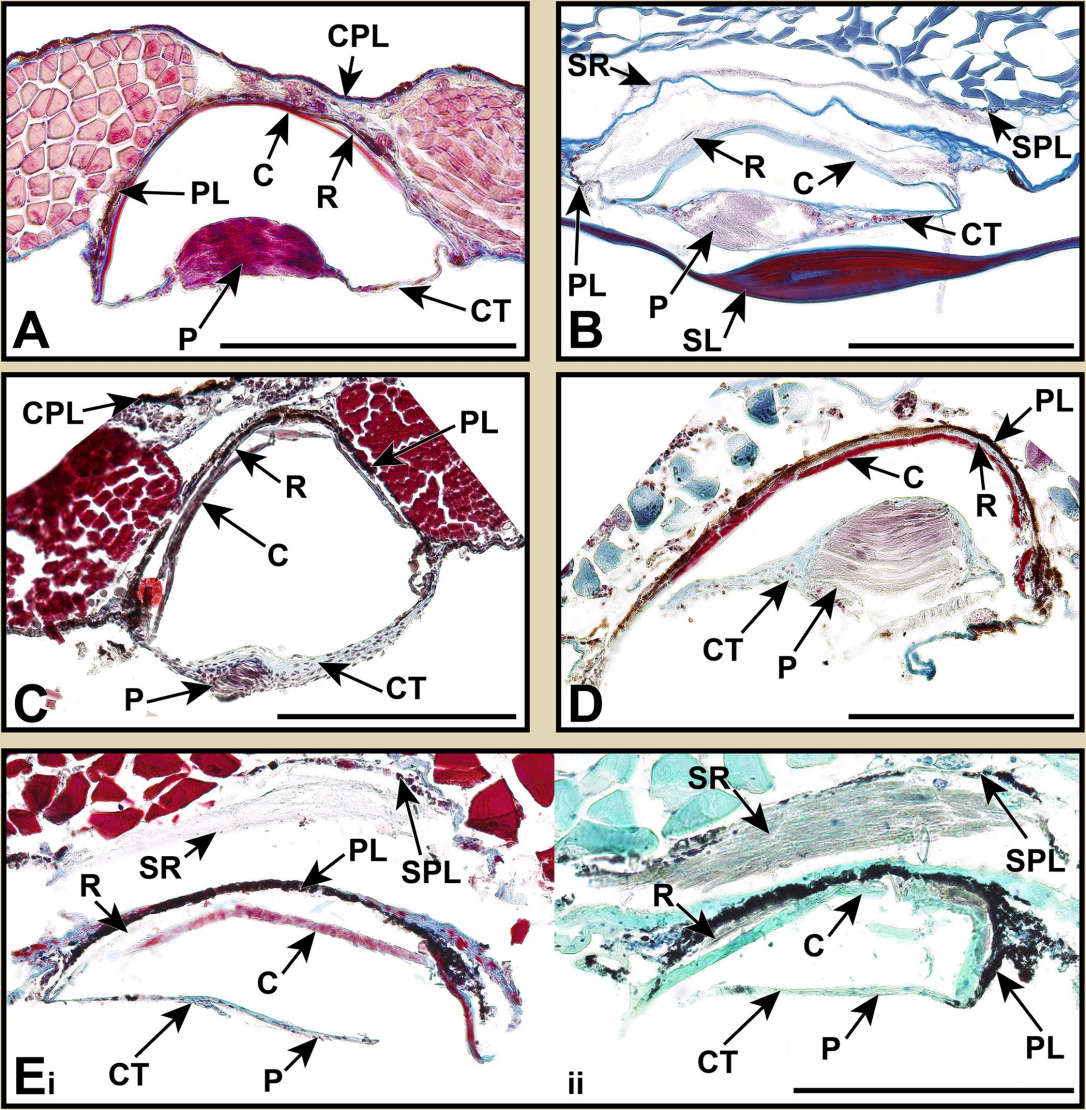
Stained sections of lanternfish primary VO photophores from species in the subfamily Myctophinae. Organized alphabetically by genus. Masson’s trichrome: (A) *Krefftichthys anderssoni* LACM 11494–12. (B) *Loweina rara* LACM 38251–3. (C) *Metelectrona ventralis* LACM 11077–13. (D) *Protomyctophum arcticum* AMNH 215743. (E_i_) *Symbolophorus evermanni* AMNH 270083. Toluidine blue: (E_ii_) *Symbolophorus evermanni* AMNH 270083. Abbreviations are as follows: C, cup; CPL, coelom pigment layer; CT, connective tissue; E, epidermis; LR, lens reflector; P, photocytes; PL, pigmented layer; R, reflector; SL, scale lens; SPL, secondary pigment layer; SR, secondary reflector. Scale bars represent 200 μm.

**Fig 8:**
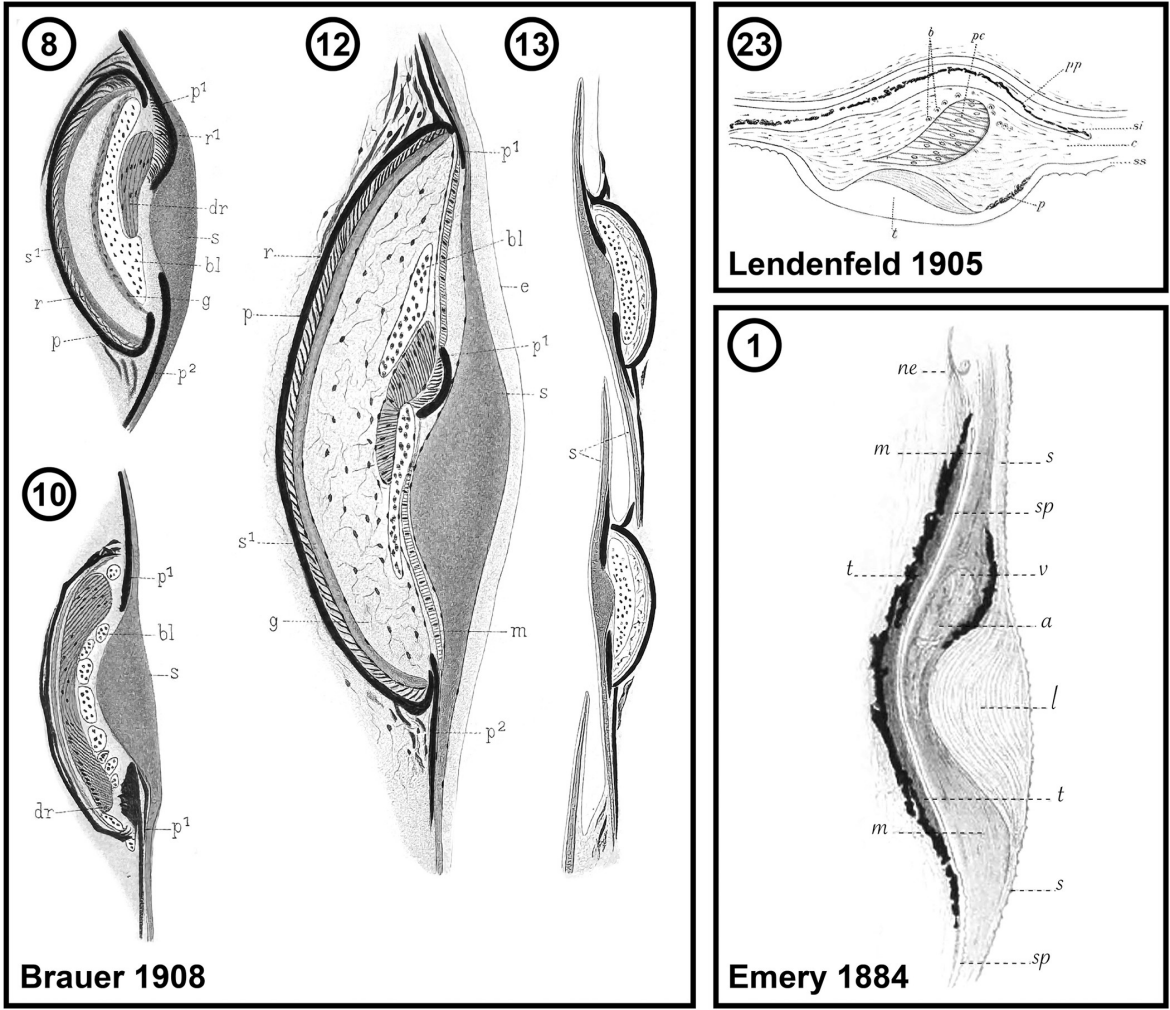
Illustrations of the morphology of lanternfish primary photophores from early previously published works. Brauer [40] Tafel XXX, Fig (8) *Hygophum benoiti*; (10) *Lampanyctus macropterus*; (12) *Diaphus dumerilii*; (13) *Bolinichthys longipes*. Abbreviations for Brauer [40] are as follows: bl, blood vessel; dr, glandular cells; e, epidermis; g, gelatinous body; m, membrane; p, pigment; r, reflector; s, scale. Lendenfeld [39], Plate 5, Fig 23) *Myctophum aurolaternatum*. Abbreviations for Lendenfeld [39] are as follows: b, blood vessels; c, connective tissue of the interior of the organ; p, section through the outer horseshoe-shaped pigment cell band; pc, radiating cells; pp, internal pigment cell layer; si, scale underlying the radiating organ; ss, scale covering the radiating organ; t, section through the H-shaped thickening of the covering scale. Emery [36], Tafel 27, Fig 1) *Notoscopelus elongatus*. Abbreviations for Emery [36] are as follows: a, specific mass of flattened cells; l, lenticular swelling of the scale; m, mucous connective tissue; s, superficial scale; sp, deep scale; t, silver carpet; v, blood vessels.

This taxonomically-comprehensive study focuses on describing primary photophore structure and comparative morphology of representative species from all 34 lanternfish genera. Except for *Scopelopsis multipunctatus*, lateral primary photophores of all myctophid species examined exhibited photocytes (P) embedded in connective tissue (CT), a pigmented mantel layer (PL), and a reflective layer (R) situated proximally to a photophore cup (C; Figs 2–8). Primary photophores of *S*. *multipunctatus* do not possess the complexity that is present in other species. Instead, photocytes appear to be embedded in a heavily pigmented epidermis and are not associated with a photophore cup or reflective layer (Fig 3E_i_-_iv_). Although the primary photophores from the VO series share many anatomical similarities across myctophid species, variation in their morphology is nevertheless readily apparent (Table 2; Figs 3–8). Specimens from many of the histological preparations generated for this study lacked one or two (*Bolinichthys longipes* [Fig 4A], *Diaphus regani* [Fig 5A], *Krefftichthys anderssoni* [Fig 7A], *Lepidophanes guentheri* [Fig 4C], *Taaningichthys minimus* [Fig 4F]) or even up to half or more (*Ceratoscopelus townsendi* [Fig 4B], *Hintonia candens* [Fig 3B], *Lampadena urophaos* [Fig 4D], *Lampanyctodes hectoris* [Fig 3C], *Lampichthys procerus* [Fig 3D], *Parvilux ingens* [Fig 4E]) of the common morphological components (i.e., photocytes, pigment layer, reflector) of the primary photophore. Species observed with half of the photophore components missing were usually damaged specimens or those that had poorly preserved photophores (Table 2 starred). Due to the inherent issues using fragile deep-sea specimens (e.g., damage during collection, old and poorly preserved museum specimens), we do not consider the lack of a particular photophore component to indicate evolutionary loss in a species. Instead, we focus on describing common anatomical patterns and unique morphologies in visible components.

The melanin-based pigmentation layer (PL) was present in most species (Table 2), but its thickness varied dramatically. Relative to photophore size, *Diaphus regani* (Fig 5A), *Ctenoscopelus phengodes* (Fig 6B), *Lampadena uropahos* (Fig 4D), and *Parvilux ingens* (Fig 4E) exhibited greater coverage and distribution of melanophores and melanosomes. Prior studies indicate that heavy pigmentation may also be present in *Diaphus dumerilii* [40], *Hygophum benoiti* [40], and *Notoscopelus elongatus* [36] (Fig 8). The photocytes of *Scopelopsis multipunctatus* (Fig 3E) were also deeply nested within a thick pigment layer. Many species possessed an additional pigmented layer (CPL) surrounding the inner coelom (cropped out of most images). While this structure is evident on some of the specimens (Figs 4D, 7A and 7C) it is not a component of the primary photophore structure.

Primary photophore reflectors (R) were visible in most species. The reflective layers of some species, including *Benthosema suborbitale* and *Symbolophorus evermanni*, were easier to distinguish after staining with toluidine blue (Figs 6A_iii_ and 7E_ii_). Reflectors were most apparent (generally thicker and more visible) in members of the subfamily Myctophinae (Table 2; Figs 6–8). Alternatively, the reflectors in primary photophores of most species in the subfamilies Gymnoscopelinae and Lampanyctinae were either thin and difficult to see or completely absent (Table 2; Figs 3, 4 and 8). Of the 17 species examined in these two subfamilies, the reflectors from *Ceratoscopelus maderensis* [32], *Gymnoscopelus braueri* (Fig 3A), *Lampadena luminosa* [40], *N*. *elongatus* [36], and *Stenobrachius leucopsarus* [29] were readily apparent. In addition to the primary reflector and pigmented layers found in most other myctophid species, *B*. *suborbitale* (Fig 6A), *Loweina rara* (Fig 7B), *Symbolophorus leucopsarus* [29], and *S*. *evermanni* (Fig 7E_i_, _ii_) also possessed a secondary layer of pigment (SPL) and a secondary reflector (SR) (Table 2). Additionally, *Parvilux ingens* (Fig 4E) and *Triphoturus mexicanus* (Fig 4G) appeared to have a secondary pigment layer (SPL) but not a secondary reflector.

Mineralization of the primary photophore cup (C) varied among lanternfishes. When apparent, most species possessed a well mineralized photophore cup (stained red), including *Benthosema suborbitale* (Fig 6A), *Bolinichthys longipes* (Fig 4A), *Diaphus dumerilii* [40], *Diaphus regani* (Fig 5A), *Diaphus watasei* [33], *Gymnoscopelus braueri* (Fig 3A), *Hygophum proximum* (Fig 6E), *Idiolychnus urolampus* (Fig 5B), *Krefftichthys anderssoni* (Fig 7A), *Lampadena urophaos* (Fig 4D), *Lampanyctodes hectoris* (Fig 3C), *Lepidophanes guentheri* (Fig 4C), *Protomyctophum arcticum* (Fig 7D), *Taaningichthys minimus* (Fig 4F), *Tarletonbeania crenularis* [30], and *Triphoturus mexicanus* (Fig 4G). However, *Ctenoscopelus phengodes* (Fig 6B), *Dasyscopelus obtusirostris* (Fig 6C), *Loweina rara* (Fig 7B), *Metelectrona ventralis* (Fig 7C), and *Parvilux ingens* (Fig 4E) exhibited primary photophore cups that lacked mineralization. A thinner collagenous layer (stained blue) was present on the proximal side of the cup (C) in most species (Figs 3–7). Cup thickness relative to primary photophore size also varied across species, with thicker cups present in *C*. *phengodes* (Fig 6B), *G*. *braueri* (Fig 3A), and *D*. *regani* (Fig 5A). Lastly, *Scopelopsis multipunctatus* (Fig 3E) lacked a photophore cup entirely.

Variation in the size of the photocyte (P) mass was apparent among lanternfishes. When visible, almost all species possessed a stacked lamellar arrangement of photocyte cells located approximately equidistant from both terminal ends of the photophore cup (Figs 3–7). Photocyte cells were usually nested within connective tissue (CT), and the approximate size of the photocyte mass varied relative to photophore size. Larger relative photocyte masses were seen in *Gymnoscopelus braueri* (Fig 3A), *Krefftichthys anderssoni* (Fig 7A), *Lampichthys procerus* (Fig 3D), *Lepidophanes guentheri* (Fig 4C), *Loweina rara* (Fig 7B), *Protomyctophum arcticum* (Fig 7D), and *Triphoturus mexicanus* (Fig 4G). The morphology of the photocyte cells in *Benthosema suborbitale* (Fig 6A_i_-_iii_) was unlike any of the other species analyzed in this study. Connective tissue was attached to what appeared to be a thick solid mass that lacked the nucleated lamellar stacking seen in other lanternfishes species. Primary photophore structure of *Hygophum proximum* (Fig 6E) is morphologically similar to *B*. *suborbitale*, however, there is still evidence of nucleated lamellar photocytes.

### Scale lens structure

We were able to histologically prepare and examine the associated modified scale lens (SL) in 10 of the 40 species across 10 genera. Previously published works that also histologically prepared the scale lens bring the total to 22 species from 17 genera [29,30,32,33,35,36,39,40]. Scale-lens morphology is characterized as having a laminated appearance consisting of long, stratified, and usually mineralized layers. The scale lens is thickened biconvexly, but the shape and depth of the arc varies among species and from distal side to proximal side (Figs 3–8). In most species, the distal side of the thickened portion of the scale lens follows the natural curvature of the rest of the scale. The tapering of the proximal side of the thickened portion was gradual in most species, including *Benthosema suborbitale* (Fig 6A_i_), *Ceratoscopelus townsendi* (Fig 4B), *Bolinichthys longipes* (Fig 4A) [40], *Diaphus dumerilii* [40], *Dasyscopelus obtusirostris* (Fig 6C), *Gymnoscopelus barueri* (Fig 3A), *Hygophum benoiti* [40], *Hygophum proximum* (Fig 6E), *Lampanyctus macropterus* [40], *Scopelopsis multipunctatus* (Fig 3E_i_), and *Triphoturus mexicanus* (Fig 4G). The transition/tapering between the thickened portion of the scale lens and the rest of the scale occurred more abruptly in *Ceratoscopelus maderensis* [32], *Ctenoscopelus phengodes* (Fig 6B), *Loweina rara* (Fig 7B), and *Notoscopelus elongatus* [36]. Overall scale-lens thickness also varied among species (Table 1; Figs 3–7), with the thickest point varying between 39.0 μm in *C*. *townsendi* (Fig 4B) and 27.7 μm in *S*. *multipunctatus* (Fig 3E_i_) to 136.1 μm in *B*. *suborbitale* (Fig 6A_i_) and 120 μm in *H*. *proximum* (Fig 6E). Although previous studies included only limited information about the scale lens of some species, most figures lack scale bars and illustrated primary photophore characteristics may not be drawn to scale e.g., [36,39,40]. Using the more recent papers that provide images with scale bars, we determine the scale lens of *C*. *maderensis* [32] to be approximately 222 μm, thicker than any of the species we prepared for this study. Additionally, the scale lens of *Diaphus watasei* [33] is approximately 88.6 μm.

In addition to differences in shape and thickness, mineralization of the scale lens also varied among lanternfish species (Figs 3–7). *Ctenoscopelus phengodes* (Fig 6B), *Gymnoscopelus braueri* (Fig 3A), *Scopelopsis multipunctatus* (Fig 3E_i_), and *Triphoturus mexicanus* (Fig 4G) possessed the most highly mineralized scale lens, with mineralization occurring throughout the entirety of the matrix and with only a thin unmineralized surface layer. Other species, including *Benthosema suborbitale* (Fig 6A_i_), *Dasyscopelus obtusirostris* (Fig 6C), and *Hygophum proximum* (Fig 6E) possessed varying degrees of mineralization on the most distal portion of the thickened area of the scale lens. In *D*. *obtusirostris*, the distal unmineralized layer was moderately thick across the entire length of the laminated area of the scale-lens with thinner extensions into the mineralized matrix (Fig 6C). A similar morphology was also observed in *H*. *proximum* but with a less well-developed distal unmineralized layer (Fig 6E). Instead of a distinct layer of unmineralized matrix, *Bolinichthys longipes* had an undefined unmineralized area within the mineralized matrix of the thickened portion of the scale lens (Fig 4A). *Benthosema suborbitale* exhibited a distinct patch of unmineralized matrix that covered approximately half of the distal surface at the midpoint of the thickened portion of the scale lens (Fig 6A_i_). This patch extended approximately one third of the way into the scale matrix. The scale lens of *Loweina rara* was unique among lanternfish species examined in that it showed reduced mineralization throughout much of the scale lens matrix (Fig 7B).

Surrounding the thick inner matrix of the scale lens, many species possessed either a thin blue stained or unstained layer. This layer was particularly visible in *Bolinichthys longipes* (Fig 3A) and *Ctenoscopelus phengodes* (Fig 6B). The scale lens of *Benthosema suborbitale* (Fig 6A_i_) had an additional layer (L?) on the most proximal side of the scale lens that stained more darkly than the mineralized matrix, which suggests that this additional layer may have a different cellular makeup than the rest of the scale lens.

## Discussion

Bioluminescence and its biochemical mechanisms, diversity of light-producing organs, and hypothesized functions has been an ongoing research topic for over a century [25,29,36,46]. Bioluminescent specializations range from simple structures, like the microstructures in the marine dinoflagellate *Pyrocystis fusiformis* [47], to complex light organs composed of transparent tissues and bioluminescent pouches [12,13] or those that secrete luminescent clouds [48]. While understanding simple bioluminescent structures is important, studying variation in complex structures, such as the primary photophores of lanternfishes, can provide insights into the evolution and diversification of bioluminescent signaling.

In this study, we find that most lanternfish species examined exhibit a general photophore morphology, which is unsurprising given that primary light organs are a synapomorphy for lanternfishes and likely evolved in their common ancestor [14,21]. However, evidence suggests lanternfishes use species-specific arrangements of primary photophores, as well as sexually dimorphic head- and tail-light organs, for intra- and interspecific communication, potentially accelerating their diversification in the deep sea [14,49,50]. Lanternfishes also possess intraocular filters that enhance their ability to visualize bioluminescent light [51]. Despite basic structural similarities in the primary photophores across Myctophidae, we nevertheless find considerable variation among species in specific aspects of primary photophore morphology. This variation may have effects on the color, intensity, and resulting luminescent signal—differences that are likely visible to conspecifics.

### Variation in primary photophore pigmentation

In deep-sea habitats, with little to no ambient light, dark pigmentation provides effective camouflage for deep-sea fishes [10]. Melanin-based pigmentation is also effective in containing and preventing the emission of bioluminescent light in unintended directions [52,53]. Lanternfishes possess pigmentation in the form of both melanophores and sheets of melanosomes, hypothesized to be useful in preventing the reflection of bioluminescent light [10]. Unsurprisingly, we find a layer of pigmentation to be a common component of the primary photophore. This layer is likely present in all species (Figs 3–8) despite being difficult to visualize in more poorly preserved specimens (e.g., *Hintonia candens* [Fig 3B] and *Lampanyctodes hectoris* [Fig 3C]).

However, the wide variation in pigmentation associated with primary photophores and scale lenses is surprising given melanosomes and melanophores are thought to occlude bioluminescent light. Leydig [35] noted similar variation and, in his description of the photophore morphology of *Diaphus rafinesquii*, remarked on how much thinner the pigmented layer was in this species than in many other individuals he examined. The most prominent and thick layers of pigmentation relative to primary photophore size occurred in *Ctenoscopelus phengodes* (Fig 6B), *Diaphus dumerilii* [40] (Fig 8), *Diaphus regani* (Fig 5A), *Hygophum benoiti* [40] (Fig 8), *Lampadena uropahos* (Fig 4D), *Notoscopelus elongatus* [36] (Fig 8), *Parvilux ingens* (Fig 4E), *Protomyctophum arcticum* (Fig 7D), and *Scopelopsis multipunctatus* (Fig 3Ei-iv).

**Fig 9:**
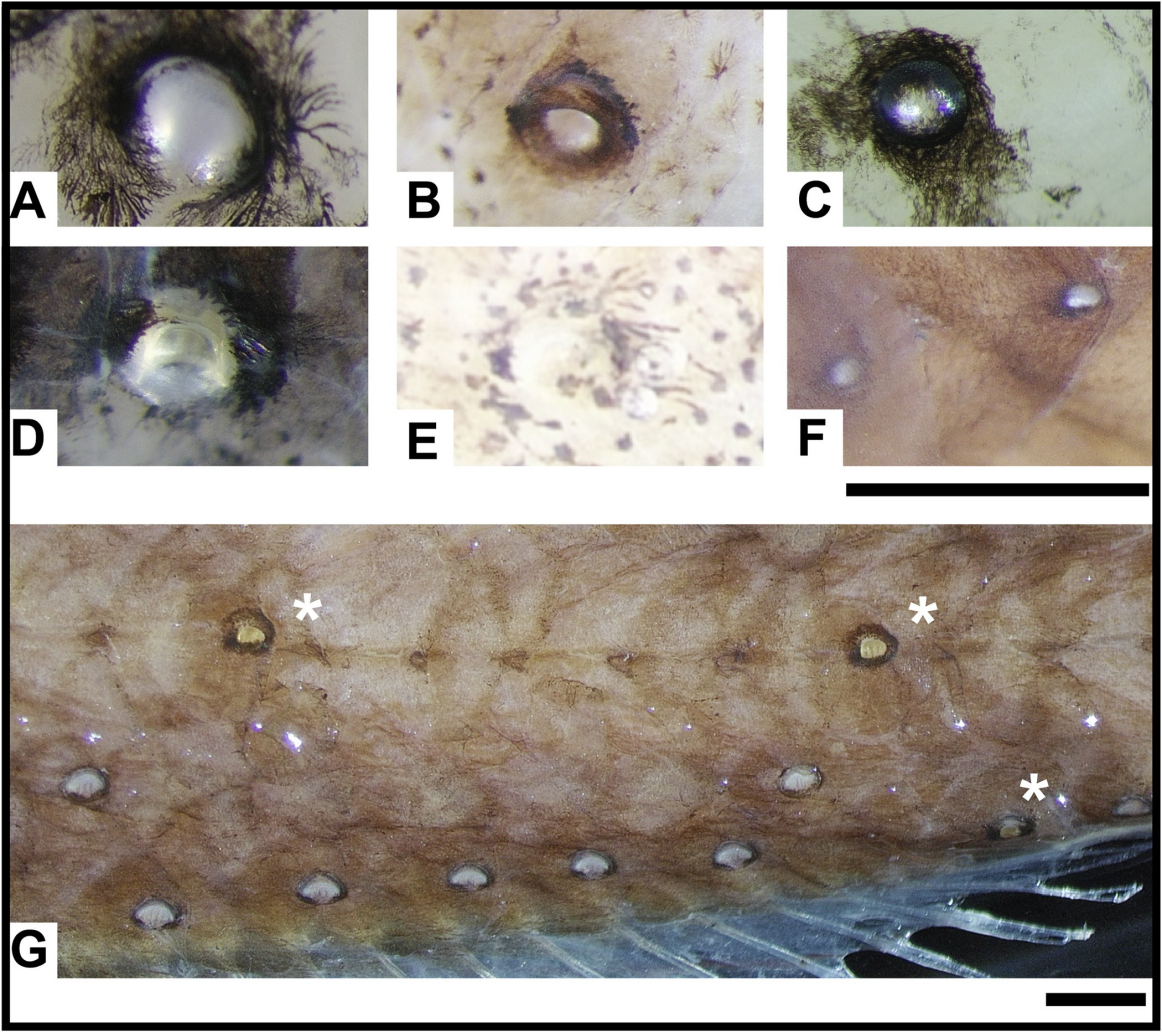
Examples of primary photophore pigment variation in formalin-fixed ethanol preserved lanternfish specimens. (A) *Benthosema suborbitale* AMNH 270136. (B) *Ctenoscopelus phengodes* LACM 31317–7. (C) *Diaphus regani* AMNH 270350. (D) *Hygophum proximum* AMNH 270116. (E) *Loweina rara* LACM 38251–3. (F) *Scopelopsis multipunctatus* LACM 31380–7. (G) Variation in photophore color in formalin-fixed ethanol preserved lanternfish specimen *Triphoturus mexicanus* (AMNH 252051) with scale lens present (starred photophores) and without the scale lens (remaining photophores). Scale bars represent 1,000 μm.

In some deep-sea fishes, melanistic pigment membranes cover the light organ, potentially acting as a diaphragm that regulates light emission and allows light to fade away [54]. Other hypotheses suggest that some bioluminescent fishes can regulate dispersion of melanocytes within melanophores surrounding their light organs [55]. Although lanternfishes appear capable of dimming their bioluminescent emissions through restricting the output from photocytes [8], it is possible that in some species, a broad layer of pigmentation across all or part of the distal surface of the photophore (e.g., as observed in *C*. *phengodes*, *D*. *regani*, *P*. *ingens*) may provide additional support to the melanocyte dispersion hypothesis (Figs 4E, 5A, 6B, 9B and 9C). Lanternfishes often possess a thicker band of pigmentation surrounding the photophore cup’s outer edge (Figs 1B and 9), likely aiding in restricting the direction of light emitted from the photophore [29].

Some lanternfish species possess a second pigment layer located deeper within the body to the first reflective layer and first pigment layer. Second pigment layers were observed in *Benthosema suborbitale* (Fig 6Ai-iii), *Loweina rara* (Fig 7B), *Parvilux ingens* (Fig 4E), and *Symbolophorus evermanni* (Fig 7Ei-ii). O’Day [29] also observed a secondary pigment layer in *Stenobrachius leucopsarus* but offered no suggestion as to its function. O’Day [29] also mentions that the first pigment layer does not always completely cover the photophore surface. Therefore, it is likely that a second pigment layer further aids in controlling or restricting the direction of emitted light by preventing its escape in unwanted directions.

### Reflectors

Numerous studies have analyzed the structure, reflectance, production, and use of guanine in fishes [56–59]. Differences in the three-dimensional organization of guanine platelets in silvery marine fishes result in the production of broadband reflectance via color mixing. These structures are hypothesized to help fish camouflage in the upper layers of the water column where, on a sunny day, wavelengths of incident light come from multiple angles [58]. The reflective layer of lanternfish primary photophores is composed of a single layer of guanine [33]. These reflective surfaces are birefringent, similar to the structure of other guanine-based iridophores [29,58].

We find that most lanternfishes possess reflectors in their primary photophores (Table 2). Based only on our histological preparations, it was difficult to determine the presence of these reflectors in most of the species in Gymnoscopelinae and Lampanyctinae (Table 2; Figs 3 and 4), whereas reflector layers were considerably more apparent in species within Myctophinae (Table 2; Figs 6 and 7). It is possible that there are differences in the characteristics of the guanified layer in species of Gymnoscopelinae and Lampanyctinae that make them thinner or affect their preservation compared to other lanternfish species. Brauer [40] describes a similar morphology in the primary photophores across most of the species he examined, but in his illustration of *Lampanyctus macropterus* (Fig 8) he does not depict the reflector layer.

Paitio et al. [33] showed that guanine iridophores of lanternfish primary photophores have a near-regular hexagonal shape, enabling interlocking geometry and one-dimensional tiling of the curved surface of the photophore. This differs from the elongated iridophores found in the tapetum lucidum (reflective layer in the eye) and the skin of lanternfishes [33]. Leydig [35] noted regular hexagonal guanine crystals in *Electrona rissoi* but less regular ones in *Myctophum punctatum*, suggesting variation in guanine crystal shape among lanternfish species within the primary photophore. Comparing the thickness and hexagonal structure of the guanified layer of fresh specimens from each subfamily may explain why this layer is absent or difficult to visualize in most gymnoscopelines and lampanyctines.

We observed secondary reflectors in several myctophid species, including *Benthosema suborbitale* (Fig 6A_i_-_iii_), *Loweina rara* (Fig 7B), and *Symbolophorus evermanni* (Fig 7E_i_-_ii_). O’Day [29] observed a similar secondary reflective layer when examining *Stenobrachius leucopsarus*. While not present in all species examined (Table 2), all four species with secondary reflectors also possessed associated secondary pigment layers (Figs 6A, 7B and 7E) [29]. We hypothesize that the presence of a secondary reflective layer and associated secondary pigment layer in these species affects both the direction and intensity of emitted luminescence.

Since bioluminescent light is reflected off the layer of guanine prior to emission from the photophore, the characteristics of the reflector have a priority effect on the spectral quality of the light emitted. Previous studies have assessed the cellular structure of lanternfish primary photophores located in both lateral and ventral positions. Paitio et al. [33] measured the wavelength of reflectance in fresh lanternfish specimens and found variation among ventral photophores. If the guanine crystals in the secondary, proximal reflective layer observed in some species of lanternfishes have a different crystalline shape, pitch, or orientation from the first, they could alter the wavelength emitted by bioluminescent light that might have penetrated the first layer, similar to the guanine platelet stacking observed in fish skin [58]. Lanternfish photocytes emit light at ~460–470 nm [60], but spectra of reflected light from the cup reflector in a ventral primary photophore in at least one species, *Diaphus watasei*, was observed to have a green-shifted emission peak at around 525–550 nm [33]. This suggests that something, presumably the guanine iridophores, is altering the wavelength of emitted light. If being able to alter the color of emitted light from the ventral primary photophores (Fig 1B teal photophores) is a general condition in lanternfishes, it would presumably allow them to better match the color of downwelling light from the surface of the ocean, effectively obscuring their silhouettes from predators below [21].

Alternatively, being able to alter the color of emitted light from the lateral primary photophores (Fig 1B: yellow photophores) may aid in inter- or intraspecific communication by allowing species to produce unique, species-specific luminescent signals. Lanternfish eyes are tuned to better visualize the common wavelengths of bioluminescent light, and species vary in their ability to visualize this light [61,62]. Additionally, some lanternfish species possess sexually dimorphic intraocular filters [51], potentially acting as long-pass filters. These filters enhance minute spectral differences between bioluminescent wavelengths, enabling individuals to take advantage of slight differences between the color of emitted bioluminescence and that of the surrounding ambient light [63]. If lanternfishes can differentiate inter- and intraspecific differences in the colors and patterns of bioluminescent light, variation in these primary photophores could be acting as a mechanism for species recognition and reproductive isolation in open-water mesopelagic environments [14,23,64].

### Scale-cup variation

Although light-organ structure varies considerably among bioluminescent fishes, the modified scale cup (C) is unique to Myctophiformes [55]. This cup provides the solid surface to which the guanified reflector layer (R) is attached. Its shape also helps determine the reflective properties of light emitted from the photophore [29,33]. The reflective layer is internal to the cup, so light must pass through the cup matrix to be reflected by the layer of guanine (Figs 3–7). Thus, the refractive indices of the modified scale cup affect the light passing through it. Any variation in the degree of mineralization could alter how light is emitted. We find that mineralization of the modified scale cup varies across Myctophidae. A well mineralized distal (to the inner area of the photophore) portion of the modified scale cup is the common condition among lanternfishes and was observed in 17 of the species examined. This characteristic was also noted in *Diaphus watasei* [33] and *Stenobrachius leucopsarus* [29]. However, in our study, *Ctenoscopelus phengodes* (Fig 6B), *Dasyscopelus obtusirostris* (Fig 6C), *Loweina rara* (Fig 7B), *Metelectrona ventralis* (Fig 7C), and *Parvilux ingens* (Fig 4E) exhibited non-mineralized modified scale cups. Assessment of scale-cup mineralization in many species from previous studies is problematic, as either it was never commented on, or the staining methods utilized did not provide information regarding differentiation of calcified material. Regardless, based on the figures, illustrations, and author accounts from these sources, it is likely that *D*. *watasei* [33], *Hygophum benoiti* [40], *S*. *leucopsarus* [29], and *Tarletonbeania crenularis* [30] possessed well-mineralized modified scale cups.

In addition to variation in scale cup mineralization, lanternfishes also exhibit variation in cup thickness and thickness of the proximal (to the inner portion of the photophore) collagenous layer. O’Day [29] described the primary photophore of *Stenobrachius leucopsarus* as having a collagenous layer that is thicker than the mineralized layer. We observed a thinner collagenous layer present on the proximal side of the cup in most species (Figs 3–7) and a thicker mineralized layer. We also observed variation in cup thickness, with thicker scale cups present in *Ctenoscopelus phengodes* (Fig 6B), *Gymnoscopelus braueri* (Fig 3A), and *Diaphus regani* (Fig 5A). Additionally, *Scopelopsis multipunctatus* (Fig 3E), a species discussed in-depth below, was unique among lanternfishes in lacking a modified scale cup entirely.

### Modified scale-lens variation

After its creation and subsequent reflectance throughout the primary photophore, light must be transmitted through the scale lens (SL) (Fig 2). Depending on the scale-lens characteristics in relation to the surrounding marine environment, light traveling through it is subject to variation in its shape and matrix components. Although the scale lens is an integral part of primary photophore anatomy, we lack basic information regarding variation in this structure because many species have deciduous scales that are lost during collection and preservation. As a result, many researchers have not assessed scale lens morphology. When it is included in a study, it is often only briefly mentioned [29,30,32,35,36,39,40,42].

Iwai and Okamura [42] describe in moderate detail some of the scale lens variation of *Tarletonbeania crenularis*, which is composed of two convex strata: a more deeply eosin-stained ‘inner layer’ 3.0–5.5 μm thick and a less well stained ‘outer layer,’ which is 2.5–4.0 μm thick [42]. Iwai and Okamura [42] state that ‘*This is rather remarkable for its compound nature which is made up of two different structures*. *Such an exquisite biconvex lens probably would act*, *as a whole*, *very efficiently*.’ Lawry [30] also analyzed the scale lens of *T*. *crenularis*. Using live specimens, removing the accessory scales overlying the scale lens resulted in the emitted luminescence changing from blue-violet to green, yellow, or white [30]. Removal of the scale lens altogether resulted in a cessation in bioluminescence, likely due to the destruction or disruption of the photocytes. In this study we were able to compare accounts of the modified scale lens of 22 lanternfish species (10 prepared in this study) from 17 genera.

Similar to the few prior analyses [29,30,32,33,35,36,39,40], we find that the scale lens exhibits a laminated appearance consisting of long, stratified, and usually mineralized layers. In all species analyzed (Table 2) the scale lens is thickened biconvexly. In most species, the distal side of the thickened portion of the scale lens follows the natural curvature of the remainder of the scale (Figs 3–8). Additionally, tapering of the proximal side of the thickened portion is usually gradual but is more abrupt in *Ceratoscopelus maderensis* [32], *Ctenoscopelus phengodes* (Fig 6B), *Loweina rara* (Fig 7B), and *Notoscopelus elongatus* [36]. Approximate modified scale-lens thickness (Table 1) varies among species (Figs 3–7), with the thinnest measuring 27.7 μm in *Scopelopsis multipunctatus* (Fig 3E_i_) and the thickest 136.1 μm in *Benthosema suborbitale* (Fig 6A_i_).

We find the differences in scale lens thickness striking, despite the possible effects of preservation, histological preparation, and location of tissue section on observed thickness. Intraspecific scale thickness often increases with specimen length [65]. Comparing our specimens of *Scopelopsis multipunctatus* and *Benthosema suborbitale*, which measured 54 mm and 36 mm standard length (SL), respectively, we find that the thickness of the modified scale lens was approximately five times greater in the smaller *B*. *suborbitale* specimen. Additionally, although *S*. *multipunctatus* is a southern hemisphere cool-water species whereas *B*. *suborbitale* is more tropical, both species are diel migrators found in surface waters at night [23]. The importance of scale protection from predation and surface water wave action is likely similar in both species, compared to some of their deeper living relatives like *Taaningichthys*, so the difference in scale-lens thickness is unusual in regard to habitat use. We were able to measure the scale-lens thickness of *Ceratoscopelus maderensis* and *Diaphus watasei* from previous studies. The scale lens of *C*. *maderensis* [32] was approximately 222 μm, thicker than any of the species we prepared for this study. However, the SL of the specimens examined in that study was not included, and their specimens were never formalin fixed or ethanol preserved. Differences in preservation technique may not allow for an accurate comparison of scale lens thickness. It seems most likely that the variation we observe in the thickness of the scale lens is related to its use in altering the intensity or wavelength of bioluminescent emissions, but additional work assessing intra- and interspecific variation in this thickness is necessary.

In addition to variation in shape and thickness, modified scale lens mineralization varied significantly among species (Figs 3–7). Fully mineralized scale lenses were observed in *Ctenoscopelus phengodes* (Fig 6B), *Gymnoscopelus braueri* (Fig 3A), *Scopelopsis multipunctatus* (Fig 3E_i_), and *Triphoturus mexicanus* (Fig 4F). Alternatively, *Benothsema suborbitale* (Fig 6A_i_), *Dasyscopelus obtusirostris* (Fig 6C), and *Hygophum proximum* (Fig 6E) exhibited varying degrees of mineralization on only the most distal portion of the thickened area of the scale lens. *Bolinichthys longipes* (Fig 4A) and *Loweina rara* (Fig 7B) had regions within the scale lens matrix that remained unmineralized. Lawy’s [30] assessment of the modified scale lens of *Tarletonbeania* depicts a similar scale lens morphology to our specimen of *B*. *suborbitale*. He remarked that the if refractive indices of the lenses and scales were greater than those of seawater they could possibly collimate or concentrate emitted light.

Biological tissues are often heterogeneous at different scales, resulting in spatial fluctuations of the refraction index that produce light scattering [66,67]. Similar to the cornea in a vertebrate eye, fish scales are one of the few transparent biological materials, and both are partially comprised of collagen. Their transparent properties are due to the suppression of light scattered by collagen via interferences induced in the collagen fibrillar network [66,67]. Variation in the thickness of the collagenous (blue stained) and mineralized areas of the modified scale lens among lanternfish species are likely creating subtle changes in the intensity and/or wavelengths of emitted bioluminescence.

### Colored filters

Paitio and Oba [55] suggest lanternfishes are the exception among deep-sea bioluminescent fishes in lacking a pigmented filter covering their light-organ surface and instead incorporate a ‘colored’ (guanified) reflector. However, almost no comparative work has been undertaken testing for the presence of filters covering the scale lens or examining its structure across the wide diversity of lanternfishes. Although previous research has not revealed any pigmented color layers between the scale lens and the reflector in lanternfishes [29,30,32,33], in *Benthosema suborbitale* (Fig 6A_i_) we detect an unidentified layer of differentially stained cells on the most proximal side of the scale lens. This layer also appears to have a different cellular makeup than the rest of the scale lens (Fig 6A_i_). Lanternfishes possess a simple squamous epithelial layer (E) over their scale lens that is visible in many of our histological sections (Figs 3A, 4A, 4G and 6E). This delicate layer of cells comes off when the scale lens is removed. The presence of colored pigments in this layer has not been explored in previous studies and requires fresh unpreserved specimens with intact scales.

A visual assessment of the modified scale lens when present (Fig 9G: starred photophores) and absent (Fig 9G: unstarred photophores) on the primary photophores of a preserved *Triphoturus mexicanus* shows the thick melanophore pigment border and reveals a slight variation in color of the photophore when the scale lens is present. Filters and photophore covers that alter the color and intensity of emitted bioluminescent light occur in numerous other deep-sea lineages. Different stomiiform species (dragonfishes, hachetfishes, etc.) possess red-pigmented, green-pigmented, or reddish-purple filters on their photophores that are hypothesized to be used in prey-illumination, intraspecific communication, and optimization of camouflage [31,55,68]. Members of the slickhead (Alepocephalidae) genus *Xenodermichthys* and the barreleye (Opisthoproctidae) genus *Opisthoproctus* possess red and purple pigmentation, respectively, in their light organs [31,69]. The bioluminescent pinecone fish, *Cleidopus gloriamaris*, possesses a reddish-orange filter over its suborbital light organs [70]. Additionally, one species of blackchin (Neoscopelidae), the sister group to lanternfishes, possesses orange-pigmented filters on its photophores [71]. The presence of colored filters on the photophores in neoscopelids indicates that this trait is present within the lineage and may be more widespread within Myctophiformes than previously believed. Although guanine-covered reflectors likely play the most important role in color alteration of emitted bioluminescent light, additional work using freshly caught and pristine specimens is needed to determine the presence of colored filters overlaying the scale lens in lanternfishes.

### Scopelopsis multipunctatus

The gross morphology of lanternfish primary photophores and their arrangement on the body appears similar across Myctophidae. Every species, with minor differences in photophore number, possesses the complete primary photophore series’ (Fig 1B). As with many biological systems, there is often an exception to the rule. In lanternfishes that exception is *Scopelopsis multipunctatus*, and this study is the first to perform a histological examination of this species’ primary photophores. Like all other lanternfishes, the primary photophores of *S*. *multipunctatus* develop in the larval stage [64]. However, by approximately 17 mm SL, larval *S*. *multipunctatus* begin developing numerous smaller secondary photophores in horizontal lines along their body in the posterior margin of every scale pocket [43]. These primary and secondary photophores are similar in size by sexual maturity (~60 mm), making it nearly impossible to distinguish the primary from the secondary photophores [64,72]. In well-preserved specimens, Wisner [23] was able to determine the scale lens over the primary photophores by eye and described it as one way to differentiate them from secondary photophores.

Inspection of our *Scopelopsis multipunctatus* specimens through a stereomicroscope resulted in similar difficulties distinguishing primary from secondary photophores (Fig 9F), akin to the issues described by other researchers [64,72]. We dissected photophores from the area that should include the VO series from two different specimens, one with and one without scales. In the specimen with scales present, we were able to verify a VO photophore via the presence of a slightly expanded modified scale lens (Fig 3E_i_). The structure of the primary photophore was greatly reduced in complexity compared to other lanternfish species. It contained deeply red-stained photocytes nested within (and almost surrounded by) a heavy band of pigmentation (Fig 3E_i_-_iv_). We found no evidence of a modified scale cup, unlike all other currently analyzed lanternfish species. Additionally, the primary photophores of *S*. *multipunctatus* bear a consistent morphology to the secondary photophores (Fig 3E_i_-_iv_). There is also evidence of a layer of blue stained connective tissue between the photocytes and the pigment layer.

Moser and Ahlstrom [64] hypothesized that the numerous secondary photophores of *Scopelopsis multipunctatus* are the ancestral condition for Myctophiformes, and that every primary photophore in other lanternfish species can be accounted for by the enlargement of specific secondary photophores on the ventral and lateral surfaces of the body. It is likely that the genetic underpinnings of photophore development are persistent throughout lanternfishes, and it is possible that a photophore-studded body was the ancestral condition. Members of *Neoscopelus* (Neoscopelidae), the sister group to lanternfishes, have ventral light organs in intervals associated with their scales and possess photophores that appear to ‘fade out gradually’ in size at the end of photophore rows [73]. This complement of photophores on the ventral surface of *Neoscopelus* is visually quite similar to the condition in *S*. *multipunctatus* and may lend support for the possession of numerous photophores as the ancestral condition for Myctophiformes.

## Conclusions

Bioluminescence appears critical to lanternfish evolution and diversification [5,14,22,51]. However, primary photophore morphology had previously been investigated in only 13% of lanternfish species, representing less than half of myctophid genera. This study aimed to expand our understanding of variation in primary photophore morphology and anatomy by analyzing species in genera where primary photophore morphology has not previously been described. We combined new findings with prior studies to compare photophore characteristics across all 34 lanternfish genera (Table 2; Figs 3–8). Although lanternfish primary photophores share many common structural components (modified scale cup, photocytes, pigment and reflector layers; Figs 3–7), we document significant novel structural variation among species.

Most analyzed species in Gymnoscopelinae and Lampanyctinae lack or have very thin reflector layers compared to the robust reflectors observed in species in Myctophinae (Table 2; Figs 3–6). Some lanternfish species possess secondary reflector backings and secondary pigment layers (i.e., *Benthosema suborbitale* [Fig 6A], *Loweina rara* [Fig 7B], *Parvilux ingens* [Fig 4D], *Stenobrachius leucopsarus* [29], *Symbolophorus evermanni* [Fig 7E_i_, _ii_], and *Triphoturus mexicanus* [Fig 4G]). We analyzed and compared the scale lens morphology of 22 different species from 17 genera, an important but often disregarded component of the primary photophore. We find major differences in scale lens thickness and patterns of mineralization, ranging from fully mineralized (i.e., *Ctenoscopelus phengodes* [Fig 6B], *Gymnoscopelus braueri* [Fig 3A], *Scopelopsis multipunctatus* [Fig 3Ei], and *T*. *mexicanus* [Fig 4G]) to species with large distal unmineralized portions composed of a thick collagenous matrix (*B*. *suborbitale* [Fig 6Ai], *Dasyscopelus obtusirostris* [Fig 6C], and *Hygophum proximum* [Fig 6E]). Lastly, we find evidence of epidermal tissue across the modified scale lens in most species and a unique differentially stained layer associated with the proximal surface of the scale lens in *B*. *suborbitale* (Fig 6A).

Future studies using fresh specimens will be crucial to determine how observed structural variation relates to variation in th function of primary photophores. These studies should focus on confirming the presence of colored epidermal pigments that may act as filters, assessing guanine crystal variation between primary and secondary reflectors and its effects on bioluminescent spectra, and determining how scale lens matrix composition affects emitted light. Lanternfishes have evolved multiple light producing structures (e.g., primary photophores, additional head and tail-light organs) that vary considerably in structure, luminescence intensity, flashing patterns, and emitted wavelengths [8,26,28,29,33]. This variation enables lanternfishes to manifest their bioluminescence in different ways. Future work incorporating phylogenetic and ecological information will help researchers understand whether photophore characteristics show any phylogenetic patterns or are associated with specific environmental pressures (e.g., variation in migration, average depth, presence of additional light organs). Research on deep-sea bioluminescence is challenging due to difficulties keeping specimens alive after capture and observed behaviors after collection may lead to inaccurate assessment given that specimens are held in an unnatural environment. Although ROV studies with advanced imaging and sampling technologies are possible, they are expensive to operate [74]. Therefore, studies using previously caught specimens remain critical for understanding these light-producing structures and their functions.

Lanternfishes are among the most abundant and diverse bioluminescent fish groups, featuring species-specific primary photophore arrangements and often additional sexually dimorphic light organs [21,23,24]. They are diversifying faster than expected given the age of their clade, similar to other deep-sea bioluminescent lineages with unique courtship displays or species-specific light producing structures [14,75]. Detailed study of light-organ morphology and anatomy across the lineage provides crucial insight into factors contributing to lanternfish diversity in the deep open ocean, an environment lacking obvious physical barriers to reproduction. The diversity of light-organ structures in Myctophidae that are capable of producing constant, low-intensity light for counterillumination [17] or unique bright flashes for predation or communication [25], underscores the importance of bioluminescence in the evolution and diversification of this species-rich assemblage.
